# 2000 years of agriculture in the Atacama desert lead to changes in the distribution and concentration of iron in maize

**DOI:** 10.1038/s41598-021-96819-1

**Published:** 2021-08-27

**Authors:** Ale Vidal Elgueta, Nathalia Navarro, Mauricio Uribe, Kevin Robe, Frédéric Gaymard, Christian Dubos, María Fernanda Pérez, Hannetz Roschzttardtz

**Affiliations:** 1 Departamento de Ecología, Pontificia Universidad Católica de Chile, 8331150 Santiago, Chile; 2 Escuela de Antropología, Pontificia Universidad Católica de Chile, Santiago, Chile; 3 Departamento de Genética Molecular y Microbiología, Pontificia Universidad Católica de Chile, 8331150 Santiago, Chile; 4 Departamento de Antropología, Universidad de Chile, 7800284 Santiago, Chile; 5 BPMP, Univ Montpellier, CNRS, INRAE, Institut Agro, Montpellier, France

**Keywords:** Plant sciences, Ecology

## Abstract

We performed a histological and quantitative study of iron in archaeological maize seeds from prehispanic times recovered from Tarapacá, Atacama Desert. Also, we examined iron distribution changes at the cell level in embryos from ancient versus new varieties of maize. Our results show a progressive decrease in iron concentration from the oldest maize to modern specimens. We interpret the results as an effect of prehispanic agriculture over the micronutrient composition of maize.

## Introduction

There is no consensus among agronomic, nutritional, and chemical sciences on the changes suffered by micronutrients such as iron, zinc, copper, and manganese in cereal crops^1^. The unequal conditions of the experiments, difficult comparisons^2^, discrepancies in statistical methods^3^, and the lack of historical data^4^ causes these contradictory positions. The controversy deepens when it comes to the iron accumulation in maize because iron is quite variable (9.6 to 63.2 mg kg^−1^) in modern maize cultivars^5^. Moreover, despite the nutritional importance of maize today, with more than 713 million tons of annual production^6^, there are limited studies on the physiological changes associated with the absence or presence of micronutrients throughout the history of maize´s agronomic production.

Intensive agriculture for thousands of years among prehispanic agricultural communities in Tarapacá, Atacama Desert (lat. 19°00´ 21°00´S), led to changes in maize morphology. Thus, our previous studies^7^ indicated that Tarapacá prehispanic farmers modified phenotypically their ancient maize. These modifications included the significant augmentation of cobs and kernels size from a mean length of 5 cm to more than 15 cm length for cobs. Kernel size augmentation ranges from 5 mm to more than 10 mm in length. Accordingly, Tarapacá maize is clearly differentiated between two phenotypic groups: a first group dated ca. 400 BC–500 AD and a later group dated ca. 500 AD to modern times (Supplementary Fig. 1). Tarapacá maize also shows a low genetic diversity caused by human selection and genetic similarities between the prehispanic and modern maize^7^.

A more recent process of plant selection has been studied in terms of nutritional quality. The Green Revolution had a negative impact in the total amount micronutrients in cereals^8–10^. Until date, the nature of these changes is still unknown. Then, the opportunity to evaluate the micronutrient distributions and accumulation directed over ancient maize could determine if all agricultural selection, indistinctive of time and techniques, results in the loss of micronutrients in cereals.

Additionally, different methods and plant models have been used to determine where micronutrients accumulate in seeds. Focusing on iron as a model, it has been shown, by a histological approach, that this essential micronutrient accumulates in vacuoles of a specific cell layer in the *Arabidopsis thaliana* mature dry embryos^11^. Iron distribution in maize has not been studied extensively. Recently, using Perls staining on maize showed that iron accumulates principally in the embryo close to the endosperm face^12^.

In this article, we used histological and quantitative methods to observe changes over iron distribution and iron concentration in archaeological and modern maize in continuous agricultural production in Tarapacá, Atacama Desert. Thus, we asked whether the loss of iron in crops could also be observed in archaeological maize, which was under a constant agriculture selection in prehispanic times.

## Results

### Dating of archaeological kernels

The kernels used in this analysis came from seven archaeological sites. All the locations are in the same area of the Atacama Desert, close to each other, in the Tarapacá region (see Material and Methods and supplementary Fig. 1.).

**Figure 1:**
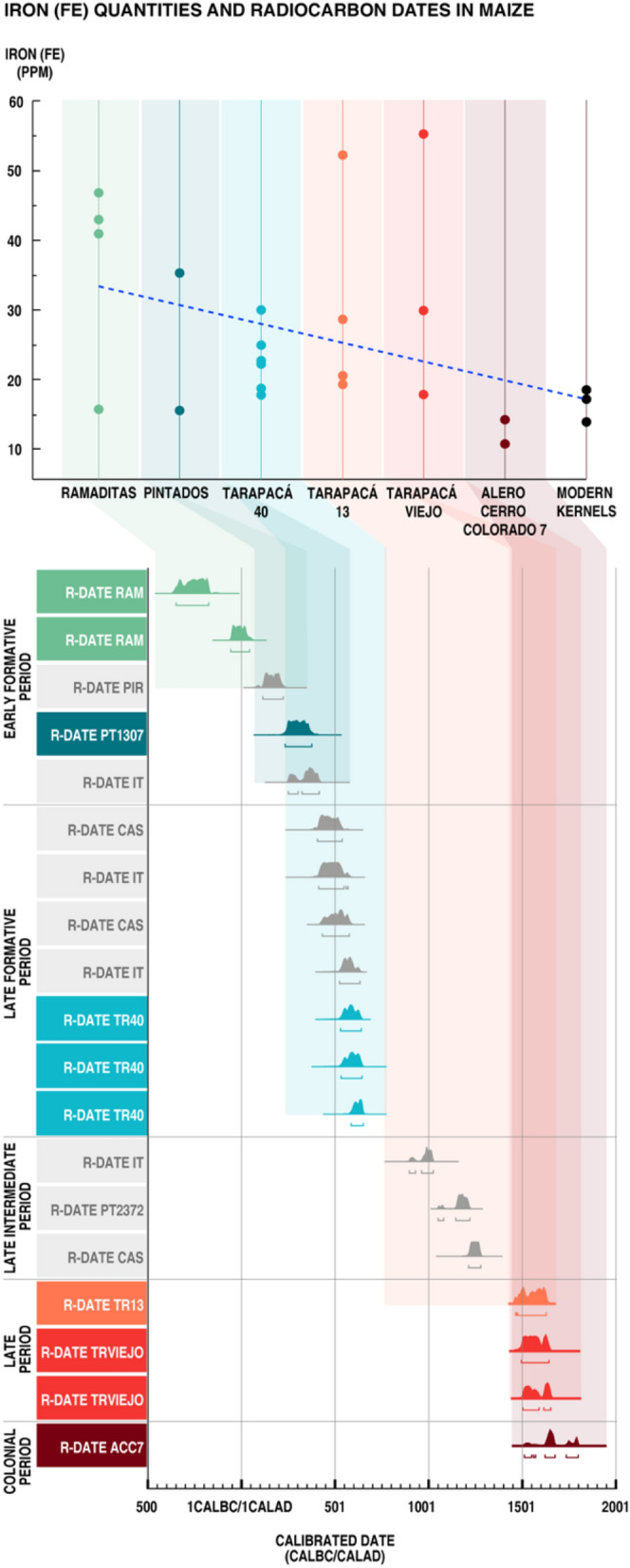
Iron (Fe) quantities and radiocarbon dates in maize. Quantifications of iron concentration in kernels are shown in color dots. Each site has been assigned to a different color and a time period. Linear regression is expressed in the dotted line. Linear regression results are: F-statistic = 4.57(1.23), R^2^ = 0.166, R^2^ adjusted = 0.129, p-value = 0.0432. The regression was calculated based on the quantities of iron in kernels and we assigned an archaeological date to each archeological site indicated in the X bar. Date was assigned as years before present. Thus, we used quantitative dates. Radiocarbon dates are indicated in color bars which are the sum of probabilities for 19 dates. Dates in color are the maize kernels for the same archaeological site used in Fe analysis. Dates in grey are reported for maize kernels from other sites for Tarapacá region. Dates range from ca. 400 BC to ca. 1799 AD.

To ensure the dates of Tarapacá maize we performed 19 radiocarbon dates directly on maize kernels and cobs from various sites of Tarapacá, including 10 dates for the samples reported in this study. The distribution of these calibrated age ranges is from 376 BC to 1799 AD (95.4% of probability) (Fig. 1 and Supplementary Table 1). This range covers a 2000 years’ time span including five different periods of the Tarapacá culture (from the Early Formative Period; ca. 1000 BC-500 AD, to Colonial Period; ca.1541–1810 AD; Fig. 1).

The most ancient sample recovered for study is a popcorn kernel from Ramaditas site, dated at 376–206 cal. BC (95.4% of probability; Fig. 1). These results show for certain that maize was already present at the southern part of Tarapacá at least from ca. 400 BC and its production continued without interruption until ca. 600 AD. Then, we noticed a disruption in maize around 600 AD to 900 AD, which could be due to a sample methodology bias, but almost for sure it is due to the temporary discontinuance of agriculture. Thus, other organic material also lacks dates for these moments. Radiocarbon dates indicate a return to agricultural maize practices around 1100–1200 AD that continues intermittently to present times (Supplementary Fig. 1).

### Iron quantification and distribution in the archaeological kernels

Quantitative analysis of iron content in individual kernels from the six archaeological sites and the modern variety is shown in Fig. 1. The kernels from Ramaditas site (376–206 cal. BC, Fig. 1) reached a high mean value of total iron concentration 36.6 ppm, while kernels from Alero Cerro Colorado 7 site (dated for seventeenth century, Fig. 1), do not exceed a mean of 13.05 ppm, corresponding to less than 36% of total iron concentration compared with the kernels from Ramaditas (Fig. 1). Our results indicate that there is a progressive decline of iron total content in the old kernels versus new varieties indicated by the relation between iron concentration in kernels and radiocarbon dates of kernels (R^2^ = 0.1297, p value < 0.043).

**Figure 2:**
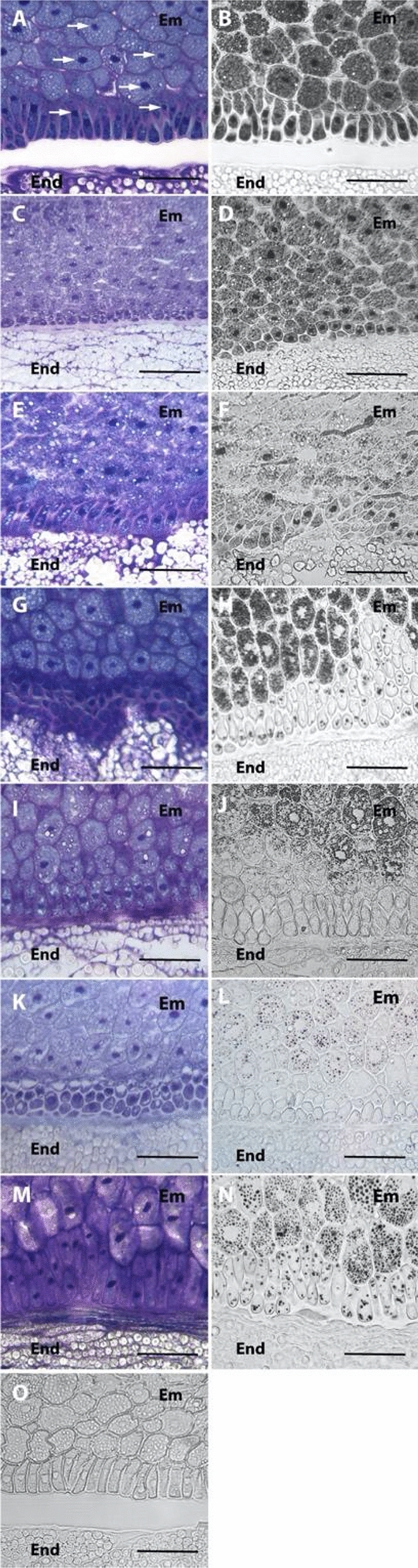
Iron distribution in kernels from archaeological sites and modern samples from Tarapacá revealed by Perls/DAB staining. (**A**–**B**,**O**) pictures from Ramaditas, (**C**,**D**) pictures from Pintados, (**E**,**F**) pictures from Tarapacá 40, (**G**,**H**) pictures from Tarapacá 13, (**I**,**J**) pictures from Tarapacá Viejo, (**K**,**L**) pictures from Alero Cerro Colorado 7, (**M**,**N**) pictures from modern sample. (**A**,**C**,**E**,**G**,**I**,**K**,**M**) correspond to toluidine blue stained sections, used to verify integrity of cells and subcellular structures in the section. (**B**,**D**,**F**,**H**,**J**,**L**,**N**) correspond to sections stained by Perls/DAB in order to detect iron, the black labeling corresponds to iron. (**O**) is a negative control of the Perls/DAB staining (without K-ferrocyanide). For all pictures, embryo and endosperm correspond to Em and End, respectively. Arrows indicate some nuclei (for simplicity only in **A**). Bars: 50 µm.

Histological sections for kernels from the six archeological sites and the modern variety were obtained and the cellular structures and iron distribution were evaluated (Fig. 2). The analysis of toluidine blue stained sections indicated that cellular structures are well preserved in all the samples, including cell wall, cytoplasmic contents and organelles like nuclei are easily identifiable. No nuclei were detected in the endosperm cells indicating that the analyzed kernels correspond to mature grains (Fig. 2A,C,E,G,I,K,M). Regarding iron localization, Perls/DAB iron staining was performed on thin sections (Fig. 2). Iron is detected in the cytoplasm and cytoplasmic structures of embryo cells in all samples, while endosperm does not accumulate detectable iron by this histological method. No staining is observed in apoplast, indicating that iron pools are intracellular in embryo. Interestingly, nuclei are strongly stained in embryo cells from older kernels (ca. 400 BC to ca. 600 AD; Fig. 2B,D,F). However, nuclear iron pools are no longer detected in embryo cells in samples from Tarapacá 13 ca. 1500 AD to actual kernels (Fig. 2H,J,L). It should be noted that the no detection of nuclear iron in the embryo cells in samples from ca. 1500 AD to actual kernels is not due to differences in the samples preservation, as indicated by the toluidine blue staining where nuclei are perfectly observable (Fig. 2G,I,K). Additionally, protodermis is less stained by Perls/DAB staining in the samples from ca. 1500 AD to actual kernels compared to the older samples (Figs. 1 and 2).

## Discussion

In this study, we showed that iron accumulates in the cytoplasm or cytoplasmic structures of maize embryo cells (Fig. 2). Interestingly, iron is not detected in nuclei of embryo cells in the kernels from ca. 1200 AD (Tarapacá 13, Tarapacá Viejo, Alero Cerro Colorado 7) to modern kernels, while iron pool is clearly observed in embryo cells from kernels older than ca. 600 AD (Ramaditas, Pintados, and Tarapacá 40; Fig. 2). Iron accumulated in nuclei has been described in the literature, but its chemical speciation and function in this organelle remain unknown^13^. It has been suggested that nuclei may be a reservoir of iron during seed development^14^. A dynamic of intracellular iron accumulation has been observed during embryo maturation stages in brassicaceae plants, however, further studies should be performed to address these fundamental biological questions about the physiological role of the intracellular iron pools^13,14^.

During the Early Formative Period (ca. 1000 BC–500 AD), the first agricultural display produced maize kernels with high contents of iron (Figs. 1 and 2). During this period, maize had a low degree of manipulation^7,15^. Thus, there is a low amount of macrobotanical maize remains in archaeological sites that do not exceed 3% of the total macrobotanicals recovered in the period’s settlements^16^. Also, isotope studies (δ^13^C and δ^15^N) in human remains from the Tarapacá 40 cemetery indicate a gradual transition to the consumption of C^4^ plants among the Tarapacá groups^17^. It is then stated that maize would not have been the main crop during the Early Formative period. Accordingly, agricultural production is extensive but not intensive. We suggest the mechanisms of artificial selection are not fully operating yet. At the end of the Late Formative Period (ca. 500–1200 AD), cultivation fields were extended to the north and south of Tarapacá^18^, kernels and cobs increased in size^7^, people are consuming maize^17^, and we observed iron loss in maize embryos (this study). The agricultural production is clearly intensified, and thus we find storage pits and debris of cobs and kernels in different prehispanic villages such as Caserones, Lluga Túmulos, among others. We postulate that the manipulation of maize is beginning to operate, with no clear consequences yet to iron distribution, but with an important decrease in iron accumulation. However, the full disclosure of human selection is recognizable during a third moment of agriculture (Late Intermediate and Late period). By then, most of agricultural production is relegated to artificial terraces, over 2500 m.a.s.l., implementing a system of terraces that depended almost exclusively on rainfall. A few settlements remain in the Pampa, such as Tarapacá Viejo, Pica 8, Lluga Túmulos, and minor sites. The presence of maize in these villages remains steady, and in some cases, there is an intensification of production. The reason for this shift in production is not yet fully known, though environmental fluctuation could be related. But despite the environmental challenges, cobs and kernels reached their major size probably due to the prolonged human selection and the use of seabird guano fertilizer^15^.

Concomitant to the human selection responsible for increase in maize size (7, Supplementary Fig. 1), our results show that iron is rarely observed in nuclei of embryo cells, and we observed low iron total amount. Therefore, the presence of iron in nuclei of maize and the large quantities of iron reported for this period, reflect a moment in which maize is not yet being manipulated, probably the first entrance of maize into the region 2400 yr. BP. Previously, it has been suggested that a "dilution effect" could be the cause of micronutrient declination, this is the mechanism by which the increase in size of dry matter in cereals is not accompanied by the increment of micronutrient^21^. Several authors agree that similar effects accompanied the development of modern cultivars^22^. Though we cannot establish the exact mechanism by which maize lost their micronutrient qualities in prehispanic times, our results suggest that a process of ancient agriculture in Tarapacá desert, which included the progressive intensification of agricultural production and selection of grain size affected maize kernels nutritional quality. It is also remarkable that a similar process has been observed as a consequence of the Green Revolution in the last 50 years^10^. It has been reported for wheat, that the introduction of semi-dwarf, high-yielding cultivars in the mid 1960’s, coincides with a progressive decrease in micronutrients contents in the grain^8^. Nonetheless, no information is available about how intensification of agricultural production has affected the micronutrients accumulation in maize kernels during the Green Revolution, and our results suggest, like for other crops, that human selection could negatively affect the nutritional quality of grains.

There are other consequences of this study. The corroboration of ancient maize varieties which naturally have large amounts of iron and the potential deciphering of Tarapacá maize genetics linked to iron presence. Then, changes in iron distribution at subcellular levels is an interesting focus of research to understand the causes of micronutrients loss in kernels maize by agriculture practices and selection. It is in this regard that the use of archaeological seeds could help to understand the collateral effects on nutritional quality of crops under long term agricultural practices, which is a crucial trait and a global challenge for worldwide agriculture. From an agronomic point of view, it seems essential to consider in the improvement programs, in addition to the total content of nutrients, the eventual changes regarding where micronutrients are accumulated in the grains, including at the subcellular level.

## Material and methods

### Ethics statement

All permissions or licenses were obtained in order to collect and to use archaeological maize samples. All methods were carried out in accordance with relevant guidelines and regulations.

### Plant samples and radiocarbon dating

Tarapacá region is situated in the northern part of Chile, and the hinterland is part of the Atacama Desert characterized as a hyper arid environment crossed by several basins. Today only two basins have permanent runoff (Tarapacá basin and Camiña basin). The archaeological sites selected for this study are all situated in the same cultural area. Thus, representing different moments of the material, social and cultural life of prehispanic communities of Tarapacá.

A total of 65 samples were analyzed. Fifty-nine archaeological maize samples were used as follows: 19 kernels were dated, 18 kernels were used for histological analysis, and 22 kernels were used for iron quantification. Additionally, 3 modern kernels were used for histological analysis, and other 3 more modern kernels were added for iron quantification. The criteria to choose these samples were the preservation of the material and the number of kernels available for each site. In some cases, samples were limited. Then, we prioritized radiocarbon dating samples. The sample belonged to the following periods and archeological sites: Early Formative Period (ca. 2000 BC-500 AD, Ramaditas), Late Formative Period (ca. 500–1200 AD, Pintados and Tarapacá 40), Late Intermediate Period (ca. 1200–1450 AD, Tarapacá 13), Late Period (ca. 1450–1541 AD, Tarapacá Viejo), Colonial Period (ca. 1520–1650 AD, Alero Cerro Colorado 7). The archaeological sites are four domestic contexts (Ramaditas, Pintados, Tarapacá 13, and Tarapacá Viejo), one funerary context (Tarapacá 40) and one ceremonial deposit (Alero Cerro Colorado 7). Archaeological kernels were cultivated locally near the sites mentioned and deposited as debris in the domestic contexts. For Tarapacá 40 and Alero Cerro Colorado 7 samples were deposited as offerings. The archaeological samples are corn types and floury types (Supplementary Fig. 1). Modern samples were collected from Camiña basin (lat. 19°18`S, lon. 69°25`W) in traditional cultivars in 2014. The modern landraces were identified as Capio Chileno and Harinoso Tarapaqueño, and one European cultivar from INRAE-France.

Radiocarbon dating was made by DirectAMS radiocarbon dating service laboratory, Seattle, Washington, USA. The samples were calibrated using the version OXCAL v4.3.2^23^ and SCHCal13 atmospheric curve^24^. All the technical parameters of the radiocarbon dating analysis are included in the Supplementary Table 1.

### Sample preparations and Perls/DAB staining

Maize kernels were longitudinally sectioned using a blade before the fixation step. The fixation and Perls/DAB staining were performed following previous studies^13,19,20^. Briefly, all samples were vacuum infiltrated with a fixation solution (2% w/v paraformaldehyde in 1 mM phosphate buffer pH 7.0) for 45 min and incubated for 16 h in the same solution. The fixated material was dehydrated with different concentration of ethanol (50%, 70%, 80%, 90%, 95% and 100%), then incubated 12 h with a solution of butanol/ethanol 1:1 (v/v), and finally incubated several hours with 100% butanol. The kernels were embedded in the Technovit 7100 resin (Kulzer) according to the manufacturer’s instructions and thin Sects. (3 µm) were obtained. For Perls/DAB staining, slides were incubated with 2% (v/v) HCl and 2% (w/v) K-ferrocyanide (Sigma Aldrich) for 45 min. For the DAB intensification, each glass slide was washed with distilled water, and incubated in a methanol solution containing 0.01 M NaN_3_ (Sigma Aldrich) and 0.3% (v/v) H_2_O_2_ (Merck) for 1 h, and then washed with 0.1 M phosphate buffer (pH 7.4). For the intensification reaction, the glass slides were incubated in 0.1 M phosphate buffer (pH 7.4) solution containing 0.025% (w/v) DAB (Sigma), 0.005% (v/v) H_2_O_2_, and 0.005% (w/v) CoCl_2_*6H2O. To stop the reaction the slides were rinsed with distilled water. The samples were observed and photographed with a Nikon Eclipse 80i microscope.

### Iron quantification

Iron quantification was performed according to Gao et al., 2020^25^. Fifteen milligrams of ground material (dry weight) per sample (one kernel) was mixed with 750 mL of nitric oxide (65% [v/v]) and 250 mL of hydrogen peroxide (30% [v/v]) prior to homogenization. Following 10 min of incubation at room temperature, the samples were mineralized using the microwave digestion systems (Berghof). Once mineralized, the nitric oxide proportion present in the samples was adjusted to 5 to 10% of the final volume by adding ultrapure water. Iron content present in the samples was then measured by microwave plasma atomic emission spectroscopy (Agilent Technologies).

## Supplementary Information

Supplementary Information 1.

Supplementary Information 2.
