# Author Correction: 2000 years of agriculture in the Atacama desert lead to changes in the distribution and concentration of iron in maize

**DOI:** 10.1038/s41598-021-98093-7

**Published:** 2021-09-08

**Authors:** Ale Vidal Elgueta, Nathalia Navarro, Mauricio Uribe, Kevin Robe, Frédéric Gaymard, Christian Dubos, María Fernanda Pérez, Hannetz Roschzttardtz

**Affiliations:** 1 Departamento de Ecología, Pontificia Universidad Católica de Chile, 8331150 Santiago, Chile; 2 Escuela de Antropología, Pontificia Universidad Católica de Chile, Santiago, Chile; 3 Departamento de Genética Molecular y Microbiología, Pontificia Universidad Católica de Chile, 8331150 Santiago, Chile; 4 Departamento de Antropología, Universidad de Chile, 7800284 Santiago, Chile; 5 BPMP, Univ Montpellier, CNRS, INRAE, Institut Agro, Montpellier, France

Correction to: *Scientific Reports* 10.1038/s41598-021-96819-1, published online 27 August 2021

The original version of this Article contained errors in the spelling of the authors Ale Vidal Elgueta, Nathalia Navarro, Mauricio Uribe, Kevin Robe, Frédéric Gaymard, Christian Dubos, María Fernanda Pérez & Hannetz Roschzttardtz which were incorrectly given as Vidal Elgueta Ale, Navarro Nathalia, Uribe Mauricio, Robe Kevin, Gaymard Frédéric, Dubos Christian, Pérez María Fernanda & Roschzttardtz Hannetz.

The original Article has been corrected.

