# Supplementary material for: 2000 years of agriculture in the Atacama desert lead to changes in the distribution and concentration of iron in maize
Source: Sci Rep. 2021 Aug 27;11:17322. doi: 10.1038/s41598-021-96819-1 (PMC8397760; doi:10.1038/s41598-021-96819-1)
Supplement: Supplementary file 1 — Supplementary Information 1. [file 41598_2021_96819_MOESM1_ESM.pdf]

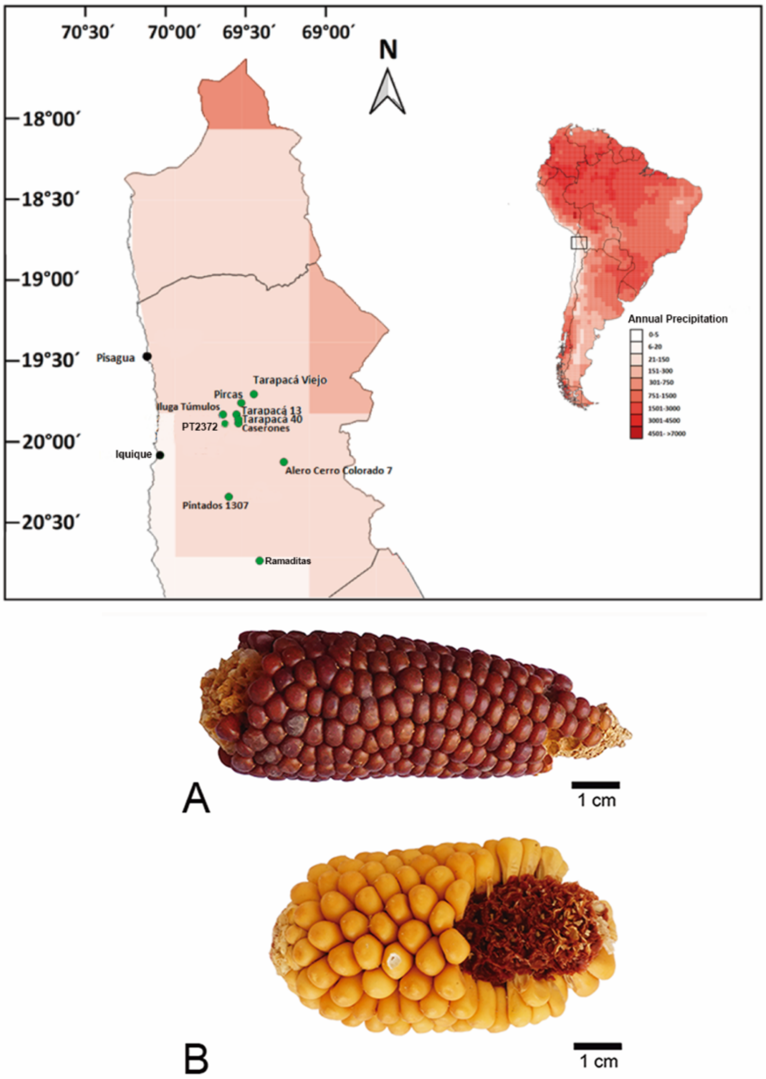

Supplementary Figure 1. Map shows the general location of the Tarapacá Region (-19°0´21°0´Lat. South) in the Atacama Desert, northern Chile, and the approximate location of the archaeological sites and Camiña. Cob A belongs to Tarapacá 40 archeological site (example of the the first group of maize), and cob B belongs to Alero Cerro Colorado 7 (example of the second group of maize). Both samples were dated (marked with an asterisk in supplementary table 1 and used for histological and Fe analysis. Images of maps obtained from QGIS 3.16.
