# Supplementary material for: 2000 years of agriculture in the Atacama desert lead to changes in the distribution and concentration of iron in maize
Source: Sci Rep. 2021 Aug 27;11:17322. doi: 10.1038/s41598-021-96819-1 (PMC8397760; doi:10.1038/s41598-021-96819-1)
Supplement: Supplementary file 2 — Supplementary Information 2. [file 41598_2021_96819_MOESM2_ESM.pdf]

Samples ID and type				Radiocarbon age		Calibrated date (BC/AD)			Fraction of modern	
DirectAMS code	ID Sample	Archaeological Site	Sample type	BP	error	from	to	%	Pmc	error
**D-AMS 033584**	**287RAMC1-B3**	**Ramaditas**	**corn kernel**	**2260**	**28**	**-376**	**-206**	**95,4**	**75.48**	**0.26**
**D-AMS 033583**	**221RAMC1-003**	**Ramaditas**	**corn kernel**	**2041**	**19**	**-56**	**45**	**95,4**	**77.56**	**0.18**
D-AMS 033582	37PIR251-3A	Pircas	corn cob	1897	19	114	224	95,4	78.96	0.19
**D-AMS 033569**	**455PT**	**Pintados**	**corn kernel**	**1778**	**30**	**234**	**376**	**95,4**	**80.14**	**0.3**
D-AMS 033573	466ITU2-4B	Iluga Túmulo	corn cob	1730	28	250	416	95,4	80.63	0.28
D-AMS 033579	591CAS526-3B	Caserones	corn kernel	1638	26	407	538	95,4	81.55	0.26
D-AMS 033571	542ITU2-2A	Iluga Túmulo	corn kernel	1619	30	413	570	95,4	81.75	0.31
D-AMS 033578	598CAS7-6B	Caserones	corn kernel	1592	24	432	577	95,4	82.02	0.25
D-AMS 033570	91ITU1-3A	Iluga Túmulo	corn kernel	1545	23	525	634	95,4	82.5	0.24
**D-AMS 033585***	**165TAR40 SR**	**Tarapacá 40**	**corn kernel**	**1522**	**32**	**530**	**640**	**95,4**	**82.74**	**0.33**
**D-AMS 033924**	**164TAR40 SR**	**Tarapacá 40**	**corn kernel**	**1533**	**27**	**533**	**645**	**95,4**	**82.63**	**0.28**
**D-AMS 033586**	**571TAR40-L-T9**	**Tarapacá 40**	**corn kernel**	**1478**	**24**	**586**	**652**	**95,4**	**83.19**	**0.24**
D-AMS 033572	1000 IT-Pto003	Iluga Túmulo	corn kernel	1109	24	897	1025	95,4	87.1	0.26
D-AMS 033568	445PT2372sup	Pintados 2372	corn cob	920	23	1051	1221	95,4	89.18	0.25
D-AMS 033577	592CAS7-5	Caserones	corn cob	830	24	1214	1279	95,4	90.18	0.27
**D-AMS 033567**	**218TAR13-A1-5**	**Tarapacá 13**	**corn kernel**	**379**	**24**	**1464**	**1629**	**95,4**	**95.39**	**0.28**
**D-AMS 033575**	**137TAR49-A8-12B**	**Tarapacá Viejo**	**corn kernel**	**352**	**25**	**1496**	**1643**	**95,4**	**95.71**	**0.3**
**D-AMS 033580**	**260TAR49-A3-7B**	**Tarapacá Viejo**	**corn kernel**	**326**	**24**	**1504**	**1653**	**95,4**	**96.02**	**0.29**
**D-AMS 033576***	**700ACC7**	**Alero Cerro Colorado 7**	**corn kernel**	**280**	**29**	**1511**	**1799**	**95,4**	**96.58**	**0.35**

Supplementary Table 1. Radiocarbon dates. Nineteen Radiocarbon dates over maize are reported. They belong to 10 archaeological sites from the Tarapacá region. Dates highlighted in bold letters with crosses were previously indicated in Figure 1 of the main text and belongs to the sites used for this study. Dates with an asterisk correspond to sample A and B presented in supplementary Figure 1. Calibration date (BC/AD) was made using OxCal v.4.3.2 (online software service) and the SHCal13 atmospheric curve.
